# Effects of 1,1,1-Trichloroethane and Triclocarban on Reductive Dechlorination of Trichloroethene in a TCE-Reducing Culture

**DOI:** 10.3389/fmicb.2017.01439

**Published:** 2017-08-03

**Authors:** Li-Lian Wen, Jia-Xian Chen, Jia-Yi Fang, Ang Li, He-Ping Zhao

**Affiliations:** ^1^Department of Environmental Engineering, College of Environmental and Resource Sciences, Zhejiang University Hangzhou, China; ^2^Zhejiang Provincial Key Laboratory of Water Pollution Control and Environmental Safety, Zhejiang University Hangzhou, China; ^3^College of Agriculture and Biotechnology, Zhejiang University Hangzhou, China; ^4^School of Environment, Harbin Institute of Technology Harbin, China

**Keywords:** trichloroethene, 1, 1, 1-trichloroethane, triclocarban, reductive dechlorination, electron distribution

## Abstract

Chlorinated compounds were generally present in the environment due to widespread use in the industry. A short-term study was performed to evaluate the effects of 1,1,1- trichloroethane (TCA) and triclocarban (TCC) on trichloroethene (TCE) removal in a reactor fed with lactate as the sole electron donor. Both TCA and TCC inhibited TCE reduction, but the TCC had a more pronounced effect compared to TCA. The TCE-reducing culture, which had never been exposed to TCA before, reductively dechlorinated TCA to 1,1-dichloroethane (DCA). Below 15 μM, TCA had little effect on the transformation of TCE to *cis*-dichloroethene (DCE); however, the reduction of *cis*-DCE and vinyl chloride (VC) were more sensitive to TCA, and ethene production was completely inhibited when the concentration of TCA was above 15 μM. In cultures amended with TCC, the reduction of TCE was severely affected, even at concentrations as low as 0.3 μM; all the cultures stalled at VC, and no ethene was detected. The cultures that fully transformed TCE to ethene contained 5.2–8.1% *Dehalococcoides*. *Geobacter* and *Desulfovibrio*, the bacteria capable of partially reducing TCE to DCE, were detected in all cultures, but both represented a larger proportion of the community in TCC-amended cultures. All cultures were dominated by *Clostridium*_sensu_stricto_7, a genus that belongs to Firmicutes with proportions ranging from 40.9% (in a high TCC (15 μM) culture) to 88.2%. *Methanobacteria* was detected at levels of 1.1–12.7%, except in cultures added with 15 and 30 μM TCA, in which they only accounted for ∼0.4%. This study implies further environmental factors needed to be considered in the successful bioremediation of TCE in contaminated sites.

## Introduction

Three typical chlorinated compounds, trichloroethene (TCE), 1,1,1- trichloroethane (TCA) and triclocarban (TCC), are common environment contaminants as a result of widespread use in industrial processes and improper disposal (Grostern and Edwards, 2006; Brausch and Rand, 2011; USEPA, 2014). TCE is classified as a human carcinogen according to a Toxic Substances Control Act (TSCA) Chemical Work Plan Chemical Risk Assessment for TCE (USEPA, 2014), and it has a maximum contaminant level (MCL) in drinking water of 5 μg/L (USEPA, 2017). TCA was banned from use and production for domestic use in United States since 2002 because it damages the ozone layer and may affect the liver, even cause death (ATDSR, 2006). The MCL of TCA in drinking water is less than 0.2 mg/L (USEPA, 2017). TCC has largely been added in detergents, soaps, cosmetics, and other personal care products at levels of 0.2–1.5% (w/w) since 1957 to inhibit microbes (Halden and Paull, 2005; Clarke and Smith, 2011; Carey et al., 2016; Souchier et al., 2016). TCC has detrimental impacts on wildlife and humans (Miller et al., 2008; Zhao J.L. et al., 2010), and the lowest effect concentration for aquatic biota is 0.101 μg/L (McClellan and Halden, 2010).

Microbially mediated anaerobic reductive dechlorination is a good strategy for the remediation of chlorinated compounds. For example, TCE can be converted to dichloroethene (DCE), vinyl chloride (VC), and finally non-toxic ethene in a stepwise manner (Lee et al., 2013; Wen et al., 2017) by various microorganisms. *Dehalococcoides* (which belong to Chloroflexi) are the only known bacteria that completely transform TCE all way down to ethene, although many other microorganisms have been found to partially reduce TCE to DCE or VC, including *Geobacter, Desulfovibrio, Desulfuromonas* (which belong to Proteobacteria), *Dehalobacter* and *Desulfitobacterium* (which belong to Firmicutes), and *Dehalobium*, and *Dehalogenimonas* (which belong to Chloroflexi) (Duhamel and Edwards, 2006; Maphosa et al., 2010).

Similarly, TCA can be transformed to 1,1-dichloroethane (DCA) and then to chloroethane (CA) via anaerobic reductive dechlorination by *Dehalobacter* or co-metabolism by *Desulfobacterium, Desulfovibrio, Clostridium*, and *Methanobacterium* (Egli et al., 1987; Gälli and McCarty, 1989). However, the degradation of TCC is quite different. Pycke et al. (2014) detected dichlorocarbanilide (DCC) and monochlorobanilide (MCC) in raw and treated sewage sludge and showed that anaerobic digestion only dechlorinated 0.4–2.1% of TCC. Souchier et al. (2016) performed field and laboratory experiments indicating that TCC reductive dechlorination occurred in anaerobic conditions to form 4,4-DCC and in aerobic circumstances to produce 3,4-DCC. Only a handful of microbial species are reportedly able to reductively reduce TCC, e.g., *Sphingomonas*, and *Ochrobactrum* (Mulla et al., 2016; Yun et al., 2017).

Trichloroethene and TCA frequently co-exist in contaminated sites due to their similar industrial uses. Data from the NPL database shows that approximately 20% of USEPA NPL sites are contaminated with both TCE and TCA. A northeastern American industrial area was polluted with 38 μM TCA and 8 μM TCE (Grostern and Edwards, 2006). Kaown et al. (2016) found that both chlorinated ethenes and ethanes were present in the industrial area of Asan, Korea, and the levels of monitored TCE and TCA ranged from 0.004 to 5.8 mg/L and from non-detected to 1.8 mg/L, respectively. Though initially was not suggested as an unidentified contamination (Halden and Paull, 2005), TCC was monitored at a value of 6750 ng/L with a frequency of 68% in United States water resources. Similarly, Zhao J.L. et al. (2010) measured 4.5–338 ng/L of TCC in the water of the Pearl River system in China and 58–2633 ng/L in its sediments. These sites were also possibly contaminated with TCE and TCA (Zhao J.L. et al., 2010; Kaown et al., 2016).

The presence of co-contaminants can greatly influence the efficiency and extent of chlorinated ethene dechlorination during *in situ* bioremediation of contaminated sites. Adamson and Parkin (2000) showed that TCA (below 20 μM) had an effect on tetrachloroethene (PCE) removal, while 10–15 μM of carbon tetrachloride (CT) inhibited the transformation of PCE and VC. Duhamel et al. (2002) found that the reduction of TCE always stalled at the step of VC conversion when the concentration of TCA was between 5.2 and 22 μM, and also stopped in the presence of 2.5 μM of chloroform (CF). Grostern and Edwards (2006) found that the presence of TCA inhibited the dechlorination of TCE and vice versa in way that was concentration independent and purely determined by the culture. Grostern et al. (2009) later suggested that chlorinated ethenes inhibited TCA dechlorination by directly affecting the reductive dehalogenase (RDase) enzymes. McDonnell and Russell (1999) reported that TCC poisoned gram-positive bacteria but had a less effect on gram-negative bacteria and fungi. Walsh et al. (2003) found that TCC was active against *Staphylococcus aureus*. Davis and Hidu (1969) reported that 0.1 μM (∼30 μg/L) of TCC caused abnormal growth of clams and reduced the survival of larvae. However, as they usually co-exist with TCE, the effects of TCA and TCC on TCE reduction remain unclear.

Dechlorinating microorganisms are always in a mixed culture, which contains many other syntrophic microbes, e.g., fermenters, methanogens and acetogens, that contribute to the rigorous nutrient requirements of the dechlorinators (He et al., 2003; Kittelmann and Friedrich, 2008; Men et al., 2013; Wen et al., 2017). Fermenters transform organic substrates, such as lactate, formate, and methanol, to acetate and hydrogen, which are used as a carbon source and electron donor, respectively, by dechlorinating microbes (Wei and Finneran, 2013; Wen et al., 2015). Homoacetogens are vital microbes that synthesize corrinoids, a significant cofactor for the growth of *Dehalococcoides* (Johnson et al., 2009; Ziv-El et al., 2012). Methanogens may also produce corrinoids for *Dehalococcoides* (Löffler et al., 1997; Wen et al., 2015). To date, TCA has been shown to inhibit methanogenesis and acetogenesis (Vargas and Ahlert, 1987; Adamson and Parkin, 2000), and TCC has been shown to inhibit methanogens and to alter microbial community structure in an anaerobic digester (Carey et al., 2016). Therefore, TCA and TCC might have negative effects on TCE reduction by affecting the microbial community structure, as they are largely consumed and always found in the environment.

The objective of this study is to explore the mechanisms behind the effects of TCC and TCA on TCE reduction. To achieve this goal, we will investigate the changes of reductive dechlorination, methane production and the electron donor distribution, along with the microbial community shifts under different conditions.

## Materials and Methods

### Description of the TCE-Dechlorinating Culture

The TCE-dechlorinating culture YH had been maintained in the laboratory for 3 years with lactate as the sole electron donor (Kranzioch et al., 2013; Wen et al., 2016). This *Dehalococcoides*-dominated culture efficiently transformed TCE (0.3 mM) to non-toxic ethene in 10 days. The culture was incubated under anaerobic conditions in dark at 30°C.

### The Effects of TCA and TCC on the TCE-Dechlorinating Culture

We prepared the anaerobic medium for TCE reduction according to Wen et al. (2015). The mineral salts medium contained the following reagents (per liter): 3.17 g KH_2_PO_4_, 14.33 g Na_2_HPO_4_ • 12H_2_O, 0.45 g (NH_4_)_2_HPO_4_, 0.04 g MgHPO_4_ • 3H_2_O, 1 mL of trace element solution A, and 1 mL of trace element solution B described by Kranzioch et al. (2013). We added 0.2 mM L-cysteine, 0.2 mM Na_2_S • 9H_2_O and 0.5 mM DL-dithiothreitol (DTT) as reducing agents, 10 mM NaHCO_3_ and 10 mM *tris*-ethanesulfonic acid (TES) as buffering agents (He et al., 2007), and 0.025% (vol/vol) resazurin as a redox indicator (Amos et al., 2008). We transferred 75 mL of medium into 120-mL glass serum bottles under a stream of argon (Ar) and then sealed the bottles with butyl rubber stoppers and aluminum crimps. We injected 0.8 mL ATCC vitamin supplement (ATCC MD-VS, United States), 80 μL vitamin B_12_ (0.5 g/L in a stock solution, and final concentration was 0.5 mg/L), 200 μL lactate (1 M in a stock solution, and final concentration was 2.5 mM) and 2.4 μl TCE (99.9% in purity, and final concentration was 0.3 mM) into the bottles with micro-syringes in an anaerobic chamber (AW200SG).

We examined the effects of TCA and TCC on TCE dechlorination separately and in combination as follows. To explore the effect of TCA on the TCE-dechlorinating culture, we added TCA to final concentrations of 0.3, 3, 15, and 30 μM in four separate bottles. Each bottle was incubated with 5 mL bacterial solution from the YH culture. To test the effect of TCC, we similarly transferred 5 mL TCE-dechlorinating culture into bottles amended with 0.3, 3, or 15 μM of TCC. To evaluate the effects of both TCA and TCC together on TCE reduction, we added both to a final concentration of 0.3 μM in the same bottle and then incubated with 5 mL TCE-dechlorinating culture. We also maintained 5 mL YH culture in a bottle with only 0.3 mM of TCE as a positive control; a bottle containing 0.3 mM TCE, 0.3 μM 1,1,1-TCA and 0.3 μM TCC in 5 mL sterile medium instead of YH culture was used as a negative control.

Each bottle was sampled periodically for TCE analysis (every 12 h during TCE reduction; every 2 days after TCE was completely reduced). All experiments were performed with duplicate bottles. The results are presented as the average values from the duplicates.

### Chemical Analysis

Chlorinated ethenes (TCE, *cis*-DCE, VC), ethene, chlorinated ethanes (TCA, DCA, CA) and methane were measured by injecting 100 μL of headspace samples with a gas-tight syringe into a gas chromatograph (Agilent Technologies GC system, model 6890N, Agilent Technologies, Inc., United States) equipped with a flame-ionization detector (FID), and a packed column (30 m long, 0.32 mm i.d., 0.5 μm thickness, cross-linked polydimethysiloxane film, J&W Scientific, United States) (Zhao H.P. et al., 2010; Ziv-El et al., 2011; Wen et al., 2017). N_2_ was the carrier gas fed at a constant flow rate of 0.065 m^3^/d, and the temperature conditions for injector and detector were 200 and 250°C, respectively. The program was as follows: holding at 60°C for 1 min, heating gradually to 200°C (20°C/min), and holding at 200°C for 2 min. Analytical grade chloroethenes, ethene, chloroethanes, and methane were added into 80 mL of water in 120 mL bottles to make standards for calibration curves, which were linear (*R*^2^ ≥ 0.996). We computed the concentrations of ethene and methane in the liquid according to their Henry’s constants (K_H_):

$${\left[\mathrm{compound}\right]}_{\mathrm{liq}}\;=\;{\left[\mathrm{compound}\right]}_{\mathrm{gas}}{/K}_{H}$$

The calculated dimensionless Henry’s constants (mM_gas_/mM_liq_, T = 25°C) used in this study were 8.35 for ethene and 28.99 for methane.

The volatile fatty acids (VFAs) lactate, acetate, and propionate were analyzed using liquid chromatography (LC, Waters) equipped with a 1525 Binary Pump, a 717 plus Autosampler, a 2487 Dual λ Absorbance Detector and an organic acid column (Acclaim^TM^ OA 5 μm, 4 mm × 250 mm). The monitored parameters were as follows: the mobile phase was 100 mM Na_2_SO_4_, the pH was adjusted to 2.65 with methylsulphonic acid (MSA), the flow rate was 0.6 mL/min, the column temperature was set at 30°C, the absorbance wavelength was 210 nm, and the injection volume was 10 μL. Liquid samples (1 mL) were filtered through a 0.22-μm polyvinylidene fluoride membrane syringe filter (Shanghai Xingya Purifying Materials Company, China) into 1 mL glass vials for subsequent analysis. Calibration curves were generated for all VFAs during every HPLC run. The detection limits for VFAs on the HPLC were 0.1 mg/L.

### Electron Distribution Analysis

The electron distribution for each reaction was calculated as described previously (Delgado et al., 2012; Wen et al., 2015). The numbers of e^-^ equivalents (eq) required for dechlorination of TCE per mole are 2, 4, and 6 to DCE, VC, and ethene, respectively, and each mole of lactate can provide 12 e^-^ eq. The electron distributions were calculated as follows:

$$\%Compound=\frac{(\mathrm{compound})\times \frac{\mathrm{electrons}}{\mathrm{mole}}}{(H_{2})\times \frac{2electrons}{\mathrm{mole}H}}\times 100$$

The related reactions and equations are listed in Supplementary Table S1.

### Molecular Biology Analysis

At the end of operation, we took 30 mL of liquid samples into 50-mL centrifuge tubes and then centrifuged for 1 h at 8000 rpm (5900 *g*) at 4°C (Eppendorf 5415R, Germany). We collected the pellets for DNA extraction as described by Zhong et al. (2017).

We used SYBR Premix Ex Taq Kits (Takara Bio, Inc., Japan) and performed qPCR amplification to target *Dhc* (for *Dehalococcoides*), *mcrA* (for methanogens), *FTHFS* (for acetogens) and the functional reductive dehalogenase genes *tceA* and *vcrA* (Wen et al., 2015). The slopes of the plasmid standard curves and efficiency values for quantification by qPCR are listed in Supplementary Table S2. We calculated gene copy numbers for biomass samples using the standard curves.

The DNA samples were sent to Novogene (Beijing, China) to perform Illumina MiSeq sequencing with standard protocols including amplicon generation, which used primers 341F (5′-CCTAYGGGRBGCASCAG-3′) and 806R (5′-GGACTACNNGGGTATCTAAT-3′) to target the conserved V3 to V4 regions of the bacterial 16S rRNA gene (Caporaso et al., 2010a), PCR products quantification, and library sequencing, which was generated using Illumina TruSeq DNA PCR-Free Library Preparation Kit (Illumina, United States) following manufacturer’s recommendations. The library quality was assessed on the Qubit@ 2.0 Flurometer (Thermo Scientific) and Agilent Bioanalyzer 2100 system and finally the library was sequenced on an Illumina HiSe platform and generated 250 bp paired-end reads. The data were processed using the QIIME (version 1.7.0) pipeline (Caporaso et al., 2010b).

## Results

### The Reductive Dechlorination of TCE in the Presence of TCC and TCA

**FIGURE 1:**
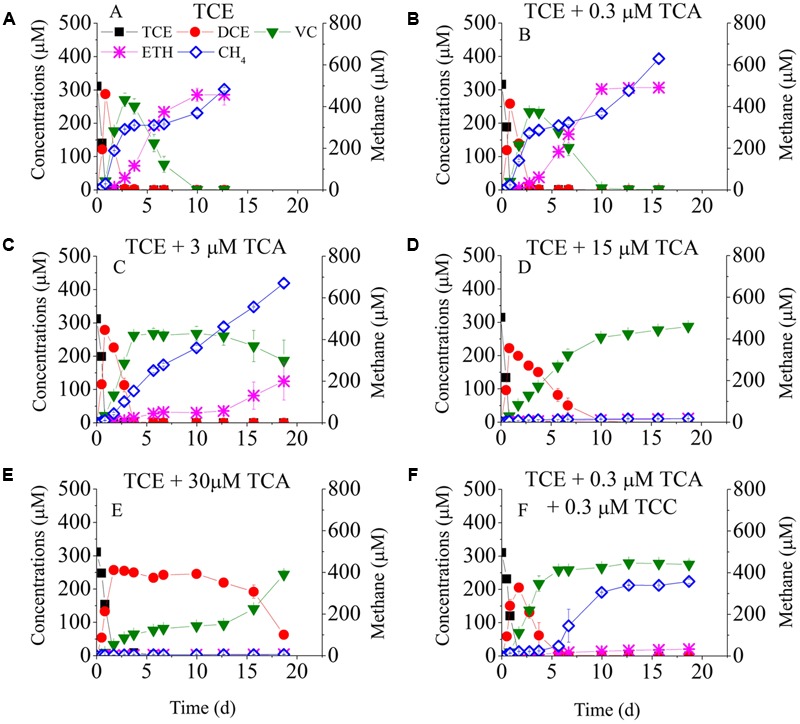
Batch tests on the dechlorination of chlorinated ethenes in the cultures exposed to different concentrations of TCA. Left *Y*-axis is the concentrations of chlorinated ethenes and ethene. Batch test **(A)** TCE only; **(B)** TCE + 0.3 μM TCA; **(C)** TCE + 3 μM TCA; **(D)** TCE + 15 μM TCA; **(E)** TCE + 30 μM TCA; **(F)** TCE + 0.3 μM TCA + 0.3 μM TCC.

**Figure 1** shows TCE reduction at different concentrations of TCA. Approximately, 0.3 μM TCE was completely reduced to ethene in 10 days at a rate of 30 μmol Cl^-^/(L-d) in the positive control batch and in the presence of 0.3 μM TCA. When the TCA concentration increased to 3 μM, same amount of TCE was mostly reduced to VC, but only 124.9 μM of ethene was detected at day 20. At concentrations of 15 and 30 μM, TCA significantly inhibited TCE reduction: *cis*-DCE was reduced to VC at day 10 and day 20, respectively, representing a delay of 6 and 16 days compared to 3 μM TCA.

**FIGURE 2:**
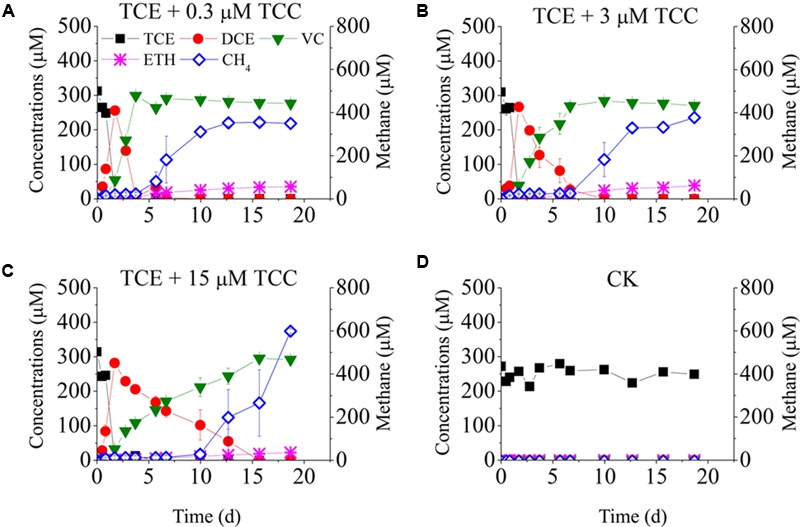
Batch tests on the dechlorination of chlorinated ethenes in the cultures amended with different concentrations of TCC. Left *Y*-axis is the concentrations of chlorinated ethenes and ethene. Batch test **(A)** TCE + 0.3 μM TCC; **(B)** TCE + 3 μM TCC; **(C)** TCE + 15 μM TCC; **(D)** Negative Control.

**Figure 2** shows TCE reduction at different concentrations of TCC. No TCE reduction was detected in the negative control. Unlike with TCA, all tested concentrations of TCC strongly inhibited TCE reduction. TCE was reduced to *cis*-DCE instantly, but the reductive rate of *cis*-DCE to VC decreased sharply, ranging from 127.9 to 20.2 μmol Cl^-^/(L⋅d) with increasing concentrations of TCC from 0.3 to 15 μM (*cis-*DCE was transformed to VC at days 4, 7, and 15 in the presence of 0.3, 3, and 15 μM TCC).

To evaluate the combined effects of TCA and TCC on TCE reduction, 0.3 μM TCA and 0.3 μM TCC were added to the cultures (**Figure 1F**). The pattern of TCE reduction was similar to that of the cultures amended with 0.3 μM TCC despite a lower dechlorinating rate, which indicated that the co-contaminants intensified the inhibition of TCE reduction.

**Figures 1, 2** also plots the models of methane production. When only TCE was added to the cultures, methane was produced rapidly at the beginning, then maintained steady from day 3 to day 7, and then increased continually to 483 μM at the end of the experiment. Low concentrations of TCA (≤3 μM) did not affect the activity of methanogens, whereas high concentrations of TCA (≥15 μM) significantly inhibited methane generation. Methanogens underwent an acclimation phase in the cultures amended with different concentrations of TCC, with 4, 7, 10 days lag when exposed to 0.3, 3, and 15 μM TCC, respectively, compared with the positive control.

Before conducting this study, the TCE-reducing culture had never been exposed to TCA. Supplementary Figure S1 shows the dechlorination of the added TCA. Approximately 0.3 μM TCA was completely removed by day 4, and no DCA and CA were detected. When the TCA concentration was increased from 3 to 30 μM, the dechlorination of TCA lagged, allowing the accumulation of intermediates DCA.

### Electron Donor Distribution in the Presence of TCC and TCA

**FIGURE 3:**
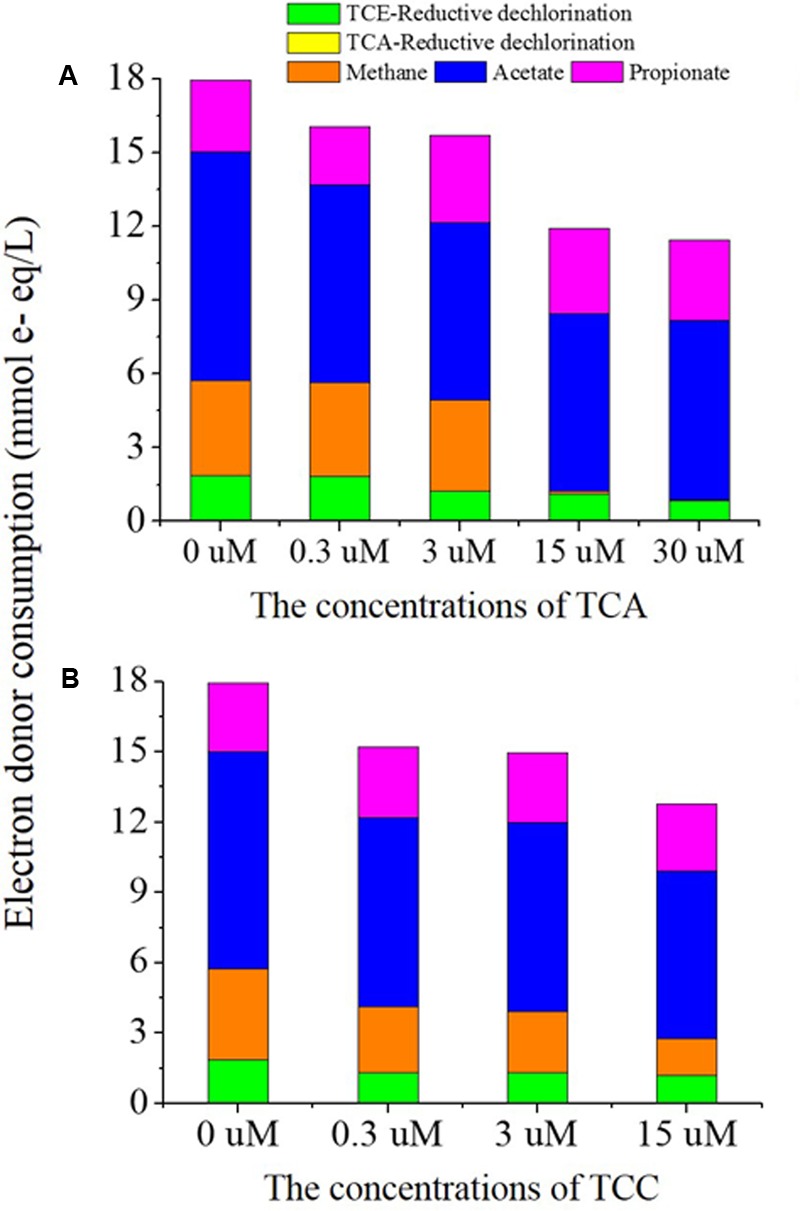
The electron donor consumption of major chemical reactions including reductive dechlorination, methanogenesis, acetogenesis and fermentation in TCA added cultures **(A)** and TCC added cultures **(B)**.

In this study, 3 mM lactate was utilized as the electron donor for all tests, which theoretically corresponds to 36 mmol e^-^ equivalents/L. In the presence of TCA, lactate was fermented to acetate and propionate instantly despite the concentration of TCA (Supplementary Figure S2). However, it took 3 days for the lactate to be fermented to acetate and propionate when TCC was present at 15 μM (Supplementary Figure S3). Considering the fermentation of lactate, we calculated the electron distribution for all dechlorination activities and methanogenesis (**Figure 3**) by taking samples at day 13, when TCE was completely reduced to ethene in the positive control batch. Most of the electron donor was consumed in the process of acetogenesis, which synthesized H_2_/HCO_3_^-^ into acetate. In the presence of 0, 0.3, 3, and 15 μM TCA, 1.87, 1.84, 1.23, 1.11, and 0.83 mmol e^-^ equivalents, respectively were distributed to reductive dechlorination of TCE; the decrease was significant for exposures ≥ 3 μM TCA. Obviously, methanogenesis did not consume any electrons because no methane was generated at concentrations of TCA ≥ 15 μM. In the presence of 0.3, 3, and 15 μM TCC, 1.31, 1.30, and 1.18 mmol e^-^ equivalents, respectively, were distributed to the reductive dechlorination of TCE. In the batch containing only TCE and no added TCA or TCC, 1.87 mmol e^-^ equivalents were directed to TCE reduction. Clearly, the addition of TCC sharply decreased the electrons distributed to TCE reduction.

### Functional Gene Abundance

**FIGURE 4:**
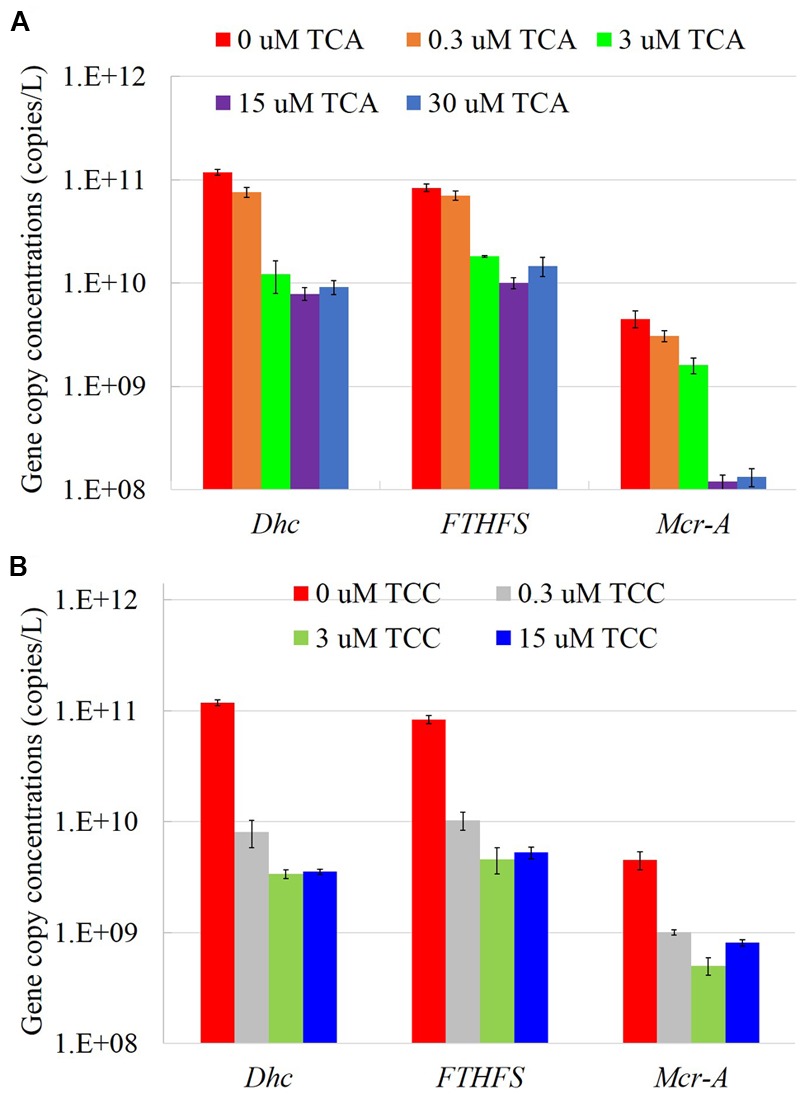
The gene copy numbers in the TCA added cultures **(A)** and TCC added cultures **(B)**.

We measured several functional genes (**Figure 4**), and dehalogenase genes (Supplementary Figure S4), in cultures amended with different electron acceptors using qPCR. In the presence of TCA, all the tested gene copies decreased with increasing concentrations of TCA. Consistent with the TCE reduction pattern, *Dhc* gene copies dropped an order of magnitude when the concentration of TCA increased to 3 μM; all tested genes abundances showed a similar pattern, except *mcrA*. When the concentration of TCA increased to 15 μM, *mcrA* gene copies decreased sharply to 1.2 × 10^8^, or 1.6 orders of magnitude lower than the positive control. In the presence of TCC, even at concentrations as low as 0.3 μM, copies of the *Dhc* and *FTHFS* genes decreased more than an order of magnitude compared to the batch containing TCE only. As shown in **Figures 1, 2**, methanogenesis was not fully inhibited in the presence of TCC, the *mcrA* gene remained at a stable abundance regardless of the concentration of TCC.

### Changes to Bacterial Community Structure

**FIGURE 5:**
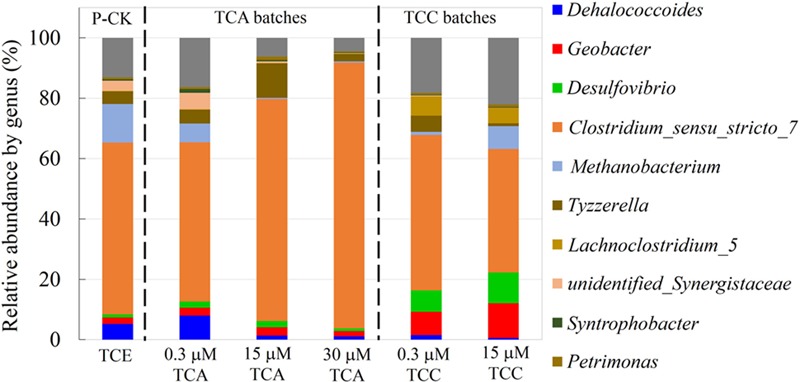
The relative abundance of microbial structure composition at the genus level in different cultures. P-CK: positive control.

We examined constituents of the microbial community at the genus (**Figure 5**) and phylum (Supplementary Figure S5) levels by Illumina MiSeq sequencing at Novogene (Beijing, China). For practical reasons, we did not send DNA samples of cultures exposed to 3 μM TCA and 3 μM TCC. In the presence of 0.3 μM TCA, *Dehalococcoides* (8.1%) was slightly higher than the positive control (5.2%). This observation was not consistent with the qPCR results, which indicated a drop in the overall abundance of *Dhc* (see Discussion). At a TCA concentration of 15 μM, the *Dehalococcodies* abundance dramatically decreased to 1%. Similarly, *Methanobacteria* decreased from 12.8% to a negligible level as the concentration of TCA increased to 30 μM. *Geobacter* and *Desulfovibrio* were present in all the cultures at stable abundances ranging from 1.0 to 2.7% regardless of the concentrations of TCA added. *Clostridium* was enriched with the addition of increasing concentrations of TCA, reaching its highest abundance of 88% in the presence of 30 μM TCA. In the presence of TCC, the proportion of *Dehalococcodies* in the community was remarkably low, dropping from 5.2% to 1.5 and 0.6% when TCC was present at 0, 0.3, and 15 μM, respectively. The abundances of *Geobacter* and *Desulfovibrio* were higher than the positive control: *Geobacter* accounted for 2.1, 7.7, and 11.5%; and *Desulfovibrio* accounted for 1.0, 7.1, and 10.2% when exposed to 0, 0.3, and 15 μM TCC, respectively. The proportion of *Methanobacteria* dramatically decreased from 12.8 to 1.1% when the culture was exposed to 0.3 μM TCC, but it increased to 7.6% in the culture exposed to 15 μM TCC, which was also reflected in the generation of methane. *Clostridium* also decreased with increasing TCC concentrations, dropping down to 48.7% in the 15 μM TCC cultures.

## Discussion

We investigated the effects of different concentrations of TCA and TCC on TCE reduction by examining the electron distribution, functional gene abundance, and bacterial community structure. This information is critical for the bioremediation of chlorinated compounds, which are generally present as co-contaminants in contaminated sites. TCA inhibited TCE reduction, previous studies demonstrated that the presence of <20 μM TCA slightly affected the removal of PCE (Adamson and Parkin, 2000). Duhamel et al. (2002) reported that VC transformation was always inhibited when the concentration of TCA ranged from 5.2 and 22 μM. We found that the complete reduction of TCE was inhibited at even lower concentrations of TCA (3 μM), and that the dechlorination of TCE stopped at the VC in the presence of ≥15 μM TCA. Grostern and Edwards (2006) suggested that the inhibition of TCA to TCE reduction was concentration independent; however, we found that 0.3 μM TCA had little effect on TCE removal. The inconsistent results may be due to the lower concentration tested in this study compared with 0.03 and 0.3 mM of TCA used in the experiment of Grostern and Edwards (2006).

Triclocarban was shown to have a more pronounced effect on TCE reduction compared with TCA that is possibly attributed to its structure with binuclear benzenes and NH_2_ or NH group, which has a detrimental effect on biodegradation (Boethling et al., 1994). To our best knowledge, this is the first report on the influence of TCC on the reductive dechlorination of TCE. TCC is reported to actively inhibit gram-positive bacteria but not gram-negative bacteria and fungi (McDonnell and Russell, 1999). Davis and Hidu (1969) reported that 0.1 μM (∼30 μg/L) TCC caused abnormal growth of clams and reduced the survival of larvae. In the TCC-amended cultures, the inhibition of TCE reduction could possibly be attributed to the toxicity of TCC to *Dehalococcoides*. Triclosan, which has a similar structure to TCC, can poison a specific enzyme that is critical to many bacteria and fungi (McMurry et al., 1998; Levy et al., 1999; Ren et al., 2010), so TCC may work analogously.

As expected in the TCE-reducing cultures, we observed the reductive dechlorination of TCA to DCA, but no CA was detected, possibly due to the short incubation period. Adamson and Parkin (2000) demonstrated that a PCE dechlorinating culture was capable of reducing TCA to DCA even without exposure before enrichment. Grostern and Edwards (2006) enriched an anaerobic culture that dechlorinated TCA to CA from a TCA-contaminated site in the northeastern United States after 70 days incubation but found that no degradation occurred in the TCE-degrading culture KB-1.

The patterns of methane and acetate production were consistent with previous reports. Methanogenesis was inhibited in the presence of ≥15 μM TCA, which might be due to the effect of intermediates of TCA reduction. TCA (<20 μM) and its daughter product DCA have been reported to inhibit methanogenesis and acetogenesis (Vargas and Ahlert, 1987; Adamson and Parkin, 2000). Grostern et al. (2009) reported no occurrence of methanogenesis in the presence of TCA, but methanogenesis started during DCA reduction. Compared to TCA, TCC had little effect on methanogenesis, which is in contrast to the results of Carey et al. (2016), who claimed that TCC inhibited methanogens and altered the anaerobic digester microbial community structure (Carey et al., 2016).

The efficient diversion of donated electrons to the process of reductive dechlorination is key to the successful removal of TCE. Hence, we further investigated the electron distribution in the presence of different concentrations of TCA and TCC. Consistent with the TCE reduction pattern, the electrons distributed to TCE reduction decreased in the presence of ≥3 μM TCA and ≥0.3 μM TCC. Usually, the methanogens are major competitors with dechlorinators (Yang and McCarty, 2002) when electron donors are supplied in excess. However, that was not the case in this study: methanogenesis did not consume more electrons when the TCA and TCC concentration increased, so TCE reduction was more likely inhibited due to reasons other than electron competition. TCA has a similar structure with TCE, which would bound to the complex formed between VC and the RDase enzyme that catalyzes growth linked dichlorination of VC (Grostern et al., 2009). Chan et al. (2011) indicated that 30–270 μg/L of 1,1,1-TCA inhibited RDases involved in TCE, *cis*-DCE, and VC dechlorination.

In general, the culture with a highest dechlorinating rate contained the maximum level of *Dhc* and reductive dehalogenases genes. In this study, the *Dhc* gene abundance significantly dropped at concentrations of TCA ≥ 3 μM and TCC ≥ 0.3 μM. Grostern et al. (2009) suggested TCA would inhibit VC reduction by binding to the complex formed between VC and the dehalogenase enzyme. The relative abundance of *Dehalococcoides* was 5.2–8.1% in the completely ethene-producing cultures, which is relatively low compared with other reports (Ziv-El et al., 2011; Löffler et al., 2013), possibly due to the lack of sufficient time to reach a higher abundance. This illustrated a higher absolute abundance of *Dehalococcoides* was important for the TCE reductive dechlorination (Lee et al., 2013).

Based on the sequencing results, the relative abundance of *Geobacter* and *Desulfovibrio* increased in cultures with higher concentrations of TCC, which indicated they were more tolerant to TCC. *Geobacter* and *Desulfovibrio*, belonging to 𝜀-Proteobacteria, are capable of reducing chlorinated organic pollutants by dehalorespiration (Smidt and de Vos, 2004). Biological reductive dechlorination of chlorinated ethenes always occurs under methanogenic conditions (Freedman and Gossett, 1989; Wen et al., 2015). Ryzhkova (2003) suggested that methanogens produce precursor corrinoids to vitamin B_12_. The abundance of *Methanobacterium* decreased when exposed to TCA and TCC, but it was less sensitive to TCC than TCA, as methanogenesis resumed after a lag phase in TCC-amended cultures. Acetogens such as Spirachaetes and *Clostridium* provide corrinoids for *Dehalococcoides* (Stupperich et al., 1988) and were present in the cultures. Zhao et al. (2008) reported that *Clostridium ganghwense* may ferment lactate to propionate. *Clostridium*_sensu_stricto_7 was highly enriched in TCA-amended cultures. Gälli and McCarty (1989) indicated that *Clostridium* sp. reduced 100 μg/L of TCA to DCA. The TCC-containing digesters had a lower fraction of *Clostridium* (Carey et al., 2016), which was consistent with the culture at a high concentration of TCC (15 μM). Many more research is needed to further understand the mechanism of the action of TCC in TCE-contaminated sites.

## Author Contributions

H-PZ designed the experiment, drafted and revised the manuscript; L-LW performed the experiment, analyzed the data, and drafted the manuscript; J-XC, J-YF, and AL helped collect the data and revise manuscript.

## Conflict of Interest Statement

The authors declare that the research was conducted in the absence of any commercial or financial relationships that could be construed as a potential conflict of interest.
