# Supplementary material for: Effects of 1,1,1-Trichloroethane and Triclocarban on Reductive Dechlorination of Trichloroethene in a TCE-Reducing Culture
Source: Front Microbiol. 2017 Aug 3;8:1439. doi: 10.3389/fmicb.2017.01439 (PMC5541058; doi:10.3389/fmicb.2017.01439)
Supplement: Supplementary file 1 [file Presentation_1.pdf]

Supplementary Information

The effects of 1,1,1-trichloroethane and triclocarban on reductive dechlorination in a TCE-reducing culture

Li-Lian Wen[1, 2], Jia-Xian Chen[1,2], Jia-Yi Fang[3], Ang Li[4], He-Ping Zhao[1, 2]*

1. Department of Environmental Engineering, College of Environmental and Resource Science, Zhejiang University, Hangzhou, China.

2. Zhejiang Prov Key Lab Water Pollut Control & Envi, Zhejiang University, Hangzhou, Zhejiang, China.

3. College of Agriculture and Biotechnology, Zhejiang University, Hangzhou, China.

4. School of Environment, Harbin Institute of Technology, Harbin, China

* Correspondance to Dr. He-Ping Zhao. Tel (Fax): 0086-571-88982739, E-mail: zhaohp@zju.edu.cn; hopechoil@hotmail.com

Table S1. Electron-donor distribution based on method of Ziv-El et al. (2012).

Steady-state end product(mM)	Biochemical Process	$\frac{me^-eq}{mmol}$	steady-state end product ($\frac{me^-eq}{L}$)
[cis-DCE],[VC],[ethene]	1) $C_2HCl_3+3H_2=C_2H_4+3Cl^-+3H^+$	6	[cis-DCE]×2+[VC]×4+[ethene]×6
	2) $C_2HCl_3+2H_2=C_2H_3Cl+2Cl^-+2H^+$	4	
	3) $C_2HCl_3+H_2=C_2H_2Cl_2+Cl^-+H^+$	2	
[DCA], [CA]	1) $C_2H_3Cl_3+2H_2=C_2H_5Cl+2Cl^-+2H^+$	4	[DCA]×2+[CA]×4
	2) $C_2H_3Cl_3+H_2=C_2H_4Cl_2+Cl^-+H^+$	2	
[Acetate]	1) $2HCO_3^- +4H_2+H^+=CH_3COO^-+2H_2O$	8	[Acetate]×8
[Methane]	1) $HCO_3^- +4H_2+H^+=CH_4+3H_2O$	8	$[CH_4]$×8
[Propionate]	1) $CH_3CH_2OCOO^-+H_2=CH_3CH_2COO^-+H_2O$	2	[Propionate]×8

Table S2. Primers and PCR conditions for tested genes

PCR Program	Primers	Sequence	Target Gene	Ref	Slope	Efficiency
95 °C 2 min (95 °C 30 sec 58 °C 30 sec 72 °C 10 sec)×30 72 °C 10 min	Dhc1200F Dhc1271R	5'-CTGGAGCTAATCCCCAAAGCT-3' 5'-CAACTTCATGCAGGCGGG-3'	*DHC*	He et al., 2003	-3.290	1.01
94 °C 10 min (94°C 30 sec 58 °C 300 sec 72 °C 60 sec)×40 72°C 1 min	Mlas rev	5'-GGTGGTGTMGGDTTCACMCARTA-3' 5'-CGTTCATBGCGTAGTTVGGRTAGT-3'	*mcrA*	Steinberg et al., 2008	-3.221	1.04
95 °C 2 min (95 °C 30 sec 58 °C 30 sec 72 °C 20 sec)×30 72 °C 10 min	fhs1 FTHFS-r	5'-GTWTGGGCWAARGGYGGMGAAGG-3' 5'-GTATTGDGTYTTRGCCATACA-3'	*FTHFS*	Xu et al., 2009	-3.442	0.95
95 °C 2 min (95 °C 30 sec 58 °C 30 sec 72 °C 10 sec)×30 72 °C 10 min	TceA1270F TceA1336R	5’-ATCCAGATTATGACCCTGGTGAA-3’ 5’-GCGGCATATATTAGGGCATCTT-3’	*TceA*	Johnson et al., 2005	-3.339	0.99
95 °C 2 min (95 °C 30 sec 58 °C 30 sec 72 °C 10 sec)×30 72 °C 10 min	Vcr1022F Vcr1093R	5’-CGGGCGGATGCACTATTTT-3’ 5’-GAATAGTCCGTGCCCTTCCTC-3’	*Vcr*	Ritalahti et al., 2006	-3.339	0.99

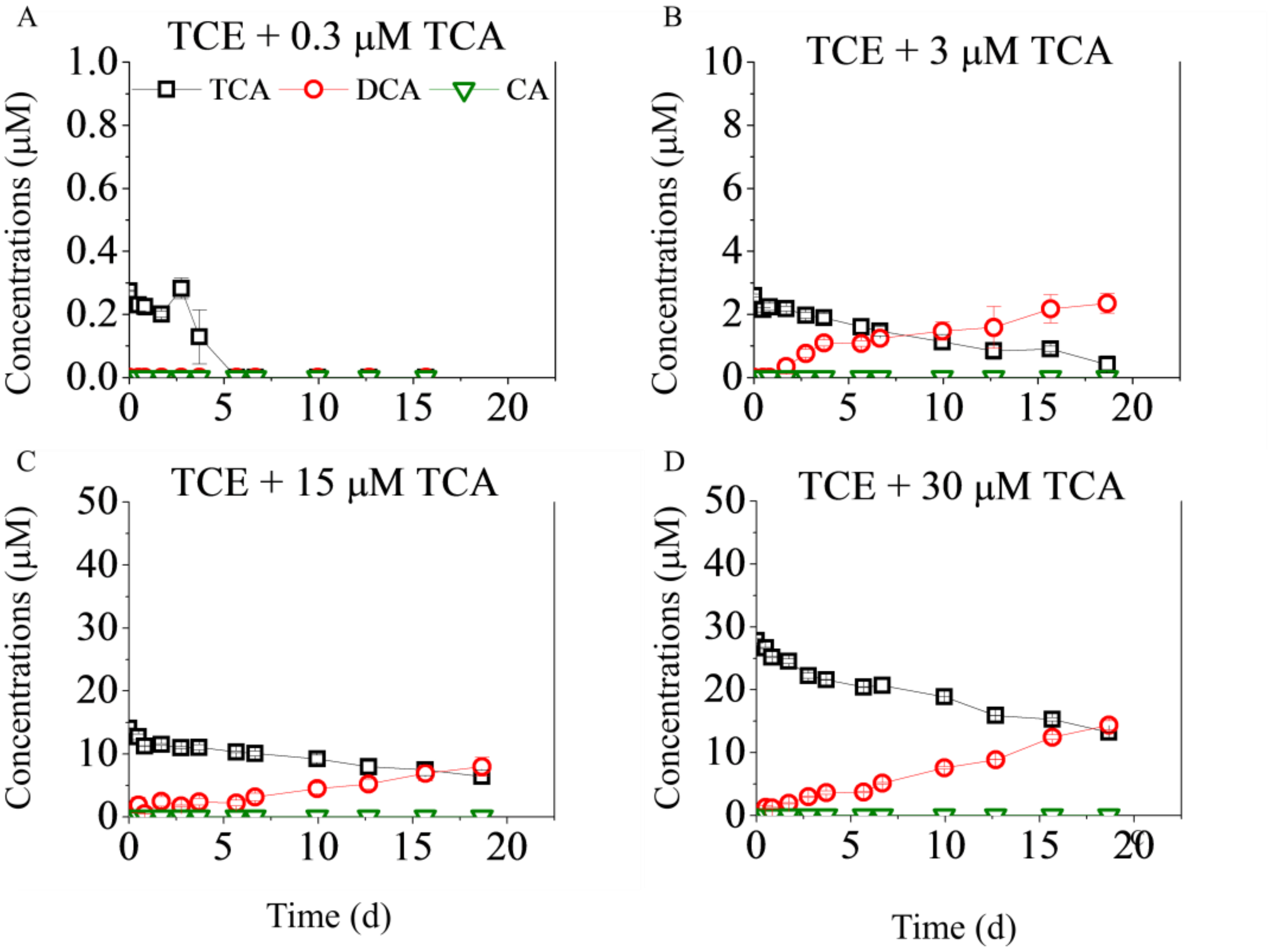

Figure S1. The reductive dechlorination of chlorinated ethanes in consortia amended with different concentrations of TCA. The left Y-axis is the concentrations of chlorinated ethanes. Batch Test A: cultures with 0.3 μM TCA added; B: cultures with 3 μM TCA added; C: cultures with 15 μM TCA added; D: cultures with 30 μM TCA added.

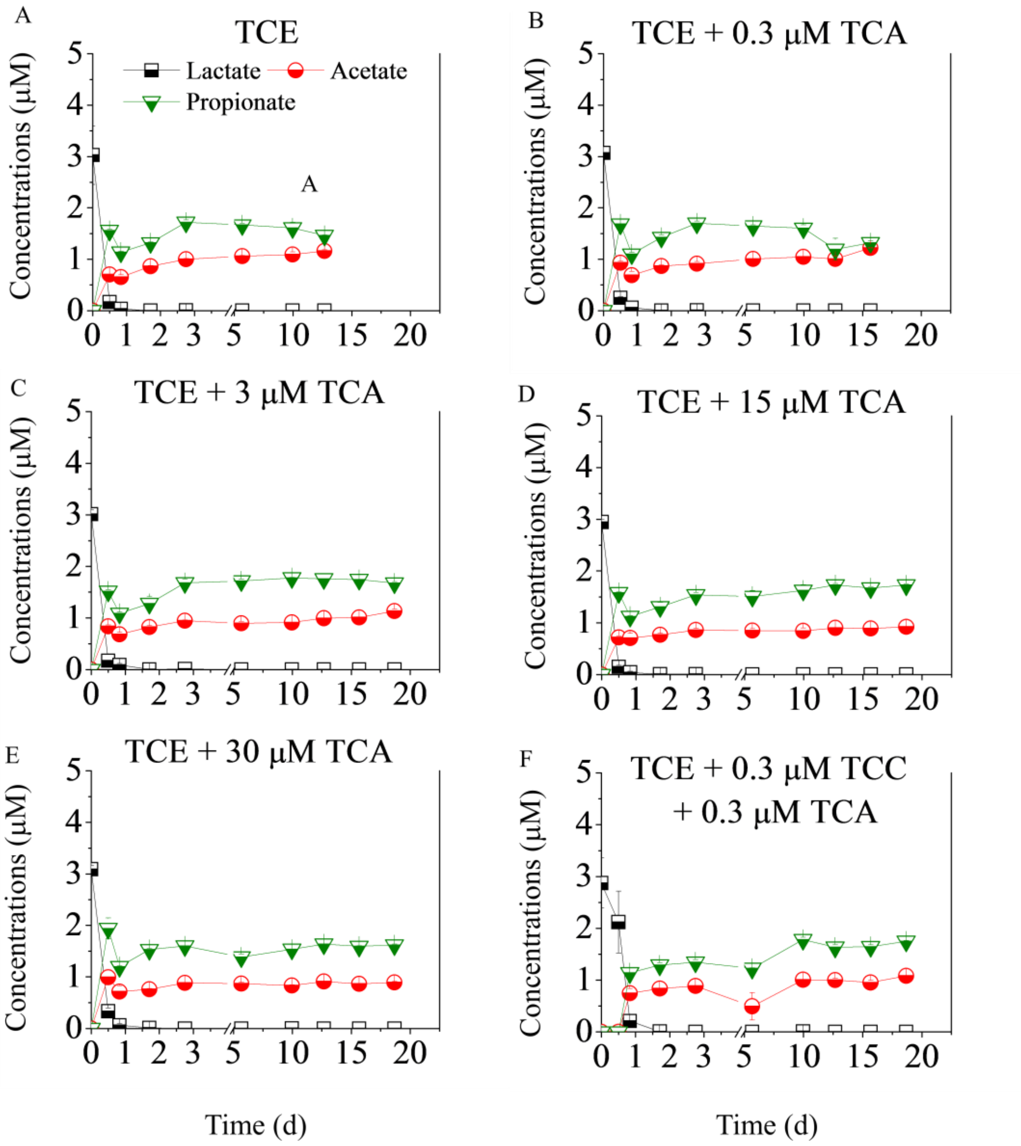

Figure S2. The concentrations of organic acids in he cultures exposed to different concentrations of TCA. The Y-axis is the concentration of volatile fatty acids. Batch test A: only TCE added; B: TCE + 0.3 μM TCA; C: TCE + 3 μM TCA; D: TCE + 15 μM TCA; E: TCE + 30 μM TCA; F: TCE + 0.3 μM TCC + 0.3 μM TCA.

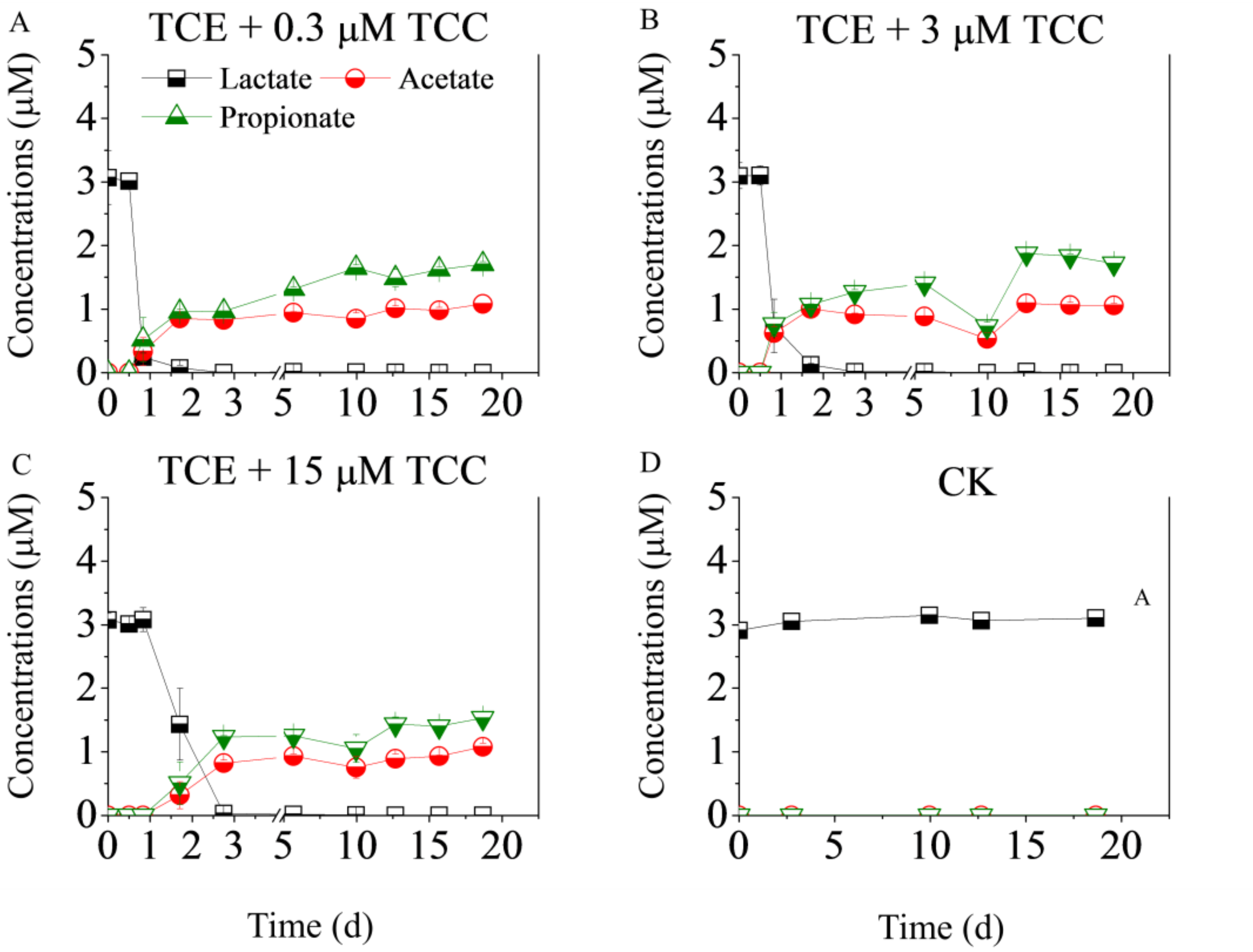

Figure S3. The concentrations of organic acids in the culture amended with different concentrations of TCC. The Y-axis is the concentration of volatile fatty acids. Batch test A: TCE + 0.3 μM TCC added; B: TCE + 3 μM TCC added; C: TCE + 15 μM TCC added; D: negative control.

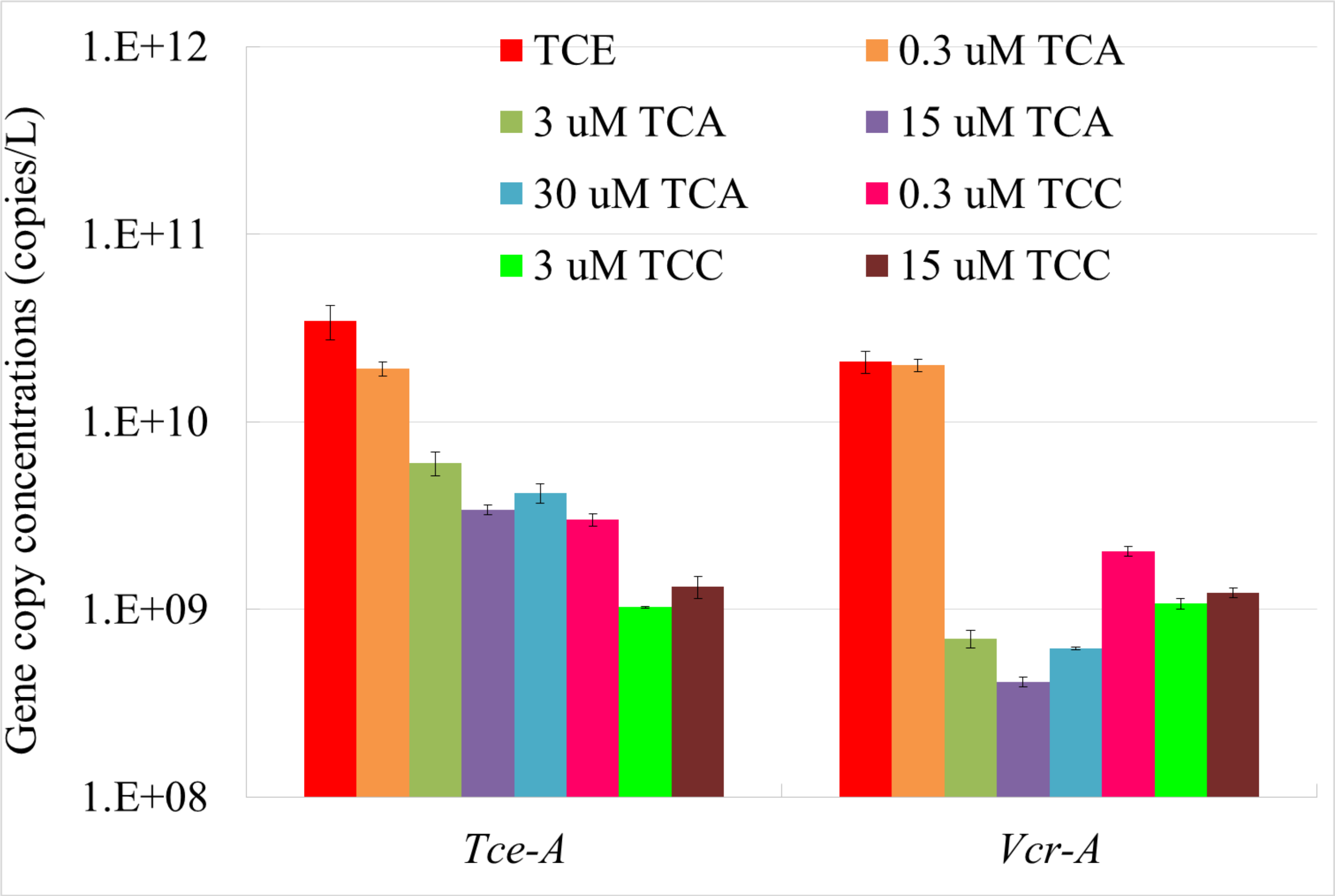

Figure S4. The concentrations of reductive dehalogenase genes copies in the culures amended with different concentrations of TCA and TCC.

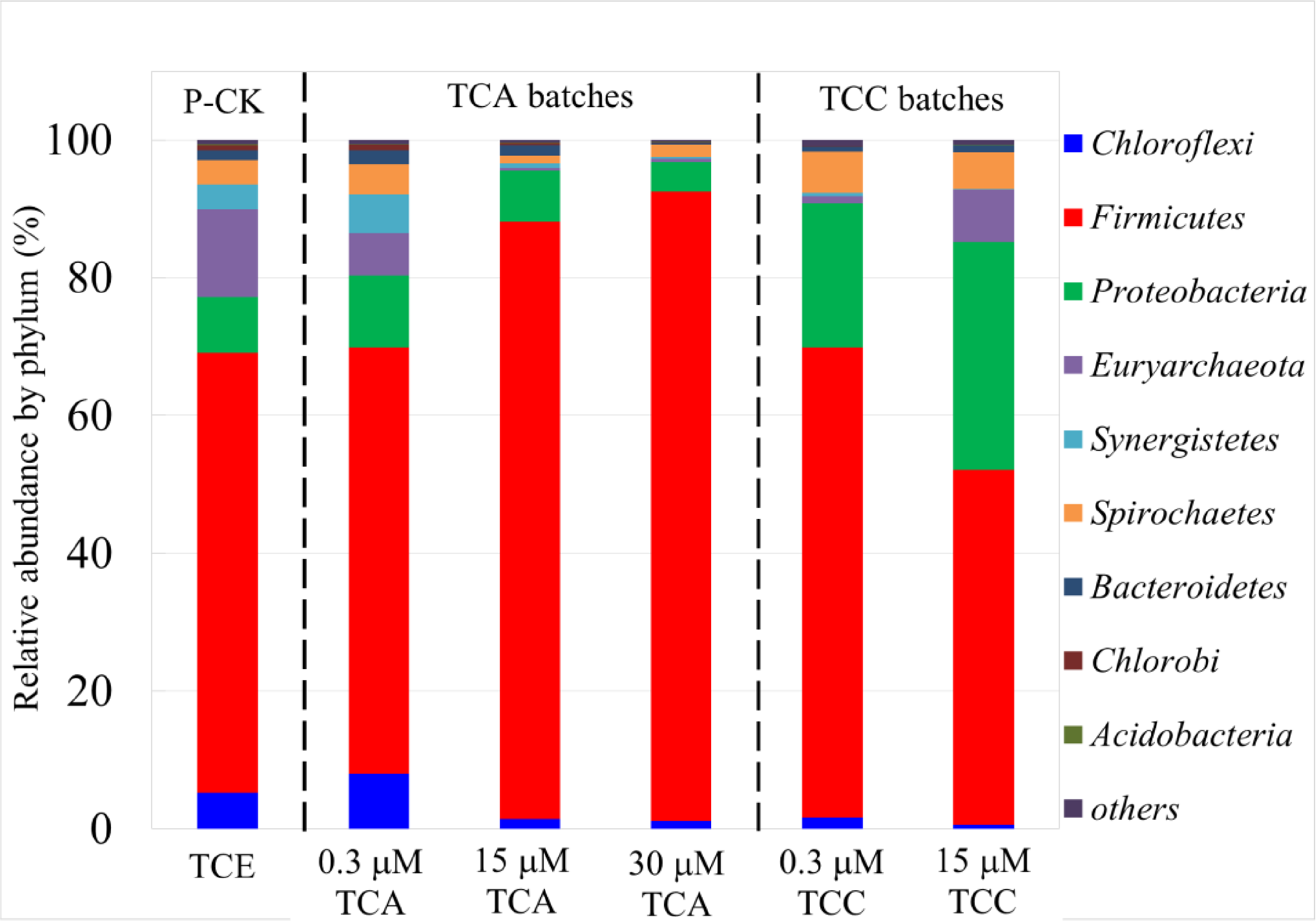

Figure S5. The relative abundance of microbial structure compositions at the phlum level in different cultures. P-CK: the culture added with 0.3 mM TCE set as a positive control; TCA batches: cultures added with 0.3, 15, 30 µM of TCA respectively; TCC batches: cultures added with 0.3, 15 µM of TCC respectively.

References

He, J., Ritalahti, K. M., Aiello, M. R., Loeffler, F. E. (2003). Complete detoxification of vinyl chloride by an anaerobic enrichment culture and identification of the reductively dechlorinating population as a *Dehalococcoides* species. *Appl. Environ. Microbiol.* 69, 996-1003.

Steinberg, L. M., Regan, J. M. (2008). Phylogenetic comparison of the methanogenic communities from an acidic, oligotrophic fen and an anaerobic digester treating municipal wastewater sludge. *Appl. Environ. Microbiol.* 74, 6663-6671.

Xu, K. W., Liu, H., Du, G. C., Chen, J. (2009). Real-time PCR assays targeting formyltetrahydrofolate synthetase gene to enumerate acetogens in natural and engineered environments. *Anaerobe.* 15, 204–213.

Johnson, D. R., Lee, P. K., Holmes, V. F., Alvarez-Cohen, L. (2005). An internal reference technique for accurately quantifying specific mRNAs by real-time PCR with application to the *tceA* reductive dehalogenase gene. *Appl. Environ. Microbiol.* 71, 3866-3871.

Ritalahti, K. M., Amos, B. K., Sung, Y., Wu, Q., Koenigsberg, S. S., Loeffler, F. E. (2006). Quantitative PCR targeting 16S rRNA and reductive dehalogenase genes simultaneously monitors multiple *Dehalococcoides* strains. *Appl. Environ. Microbiol.* 72, 2765-2774.
